# Invasive mouse gastric adenocarcinomas arising from Lgr5+ stem cells are dependent on crosstalk between the Hedgehog/GLI2 and mTOR pathways

**DOI:** 10.18632/oncotarget.7182

**Published:** 2016-02-03

**Authors:** Li-Jyun Syu, Xinyi Zhao, Yaqing Zhang, Marina Grachtchouk, Elise Demitrack, Alexandre Ermilov, Dawn M. Wilbert, Xinlei Zheng, Ashley Kaatz, Joel K. Greenson, Deborah L. Gumucio, Juanita L. Merchant, Marina Pasca di Magliano, Linda C. Samuelson, Andrzej A. Dlugosz

**Affiliations:** ^1^ Department of Dermatology, University of Michigan, Ann Arbor, MI, USA; ^2^ Department of Surgery, University of Michigan, Ann Arbor, MI, USA; ^3^ Department of Molecular and Integrative Physiology, University of Michigan, Ann Arbor, MI, USA; ^4^ Department of Pathology, University of Michigan, Ann Arbor, MI, USA; ^5^ Department of Cell and Developmental Biology, University of Michigan, Ann Arbor, MI, USA; ^6^ Department of Internal Medicine, University of Michigan School of Medicine, Ann Arbor, MI, USA

**Keywords:** gastric cancer, stem cells, Lgr5, GLI2, mTOR

## Abstract

Gastric adenocarcinoma is the third most common cause of cancer-related death worldwide. Here we report a novel, highly-penetrant mouse model of invasive gastric cancer arising from deregulated Hedgehog/Gli2 signaling targeted to Lgr5-expressing stem cells in adult stomach. Tumor development progressed rapidly: three weeks after inducing the Hh pathway oncogene GLI2A, 65% of mice harbored in situ gastric cancer, and an additional 23% of mice had locally invasive tumors. Advanced mouse gastric tumors had multiple features in common with human gastric adenocarcinomas, including characteristic histological changes, expression of RNA and protein markers, and the presence of major inflammatory and stromal cell populations. A subset of tumor cells underwent epithelial-mesenchymal transition, likely mediated by focal activation of canonical Wnt signaling and Snail1 induction. Strikingly, mTOR pathway activation, based on pS6 expression, was robustly activated in mouse gastric adenocarcinomas from the earliest stages of tumor development, and treatment with rapamycin impaired tumor growth. GLI2A-expressing epithelial cells were detected transiently in intestine, which also contains Lgr5+ stem cells, but they did not give rise to epithelial tumors in this organ. These findings establish that deregulated activation of Hedgehog/Gli2 signaling in Lgr5-expressing stem cells is sufficient to drive gastric adenocarcinoma development in mice, identify a critical requirement for mTOR signaling in the pathogenesis of these tumors, and underscore the importance of tissue context in defining stem cell responsiveness to oncogenic stimuli.

## INTRODUCTION

Gastric cancer is the 5^th^ most common cancer worldwide and the 3^rd^ most common cause of cancer-related death [1]. Although the incidence of gastric cancer in the US is relatively low due to the identification and treatment of *Helicobacter pylori*, a major risk factor [2], the 5-year survival rate for patients diagnosed with this malignancy is only 29% [3]. In contrast to multi-step tumorigenesis in colorectal or pancreatic cancer, where neoplastic progression is driven by the accumulation of genetic alterations that have been experimentally verified to contribute to tumorigenesis, the genetic basis of most gastric cancers has not been established. While somatic mutations have been detected in multiple genes encoding oncoproteins or tumor suppressors in gastric cancer, most of these are present in a subset of tumors [4-6], underscoring the molecular heterogeneity of these tumors.

Genetic mouse models have yielded insights into signaling alterations and effector molecules that can contribute to gastric tumor development. Prominent among these are models designed to mimic alterations in pro-inflammatory cytokines and signaling pathways that are deregulated in human gastric cancer, including IL-1β; IFNγ; Wnt/β-catenin; Cox2 and prostaglandin E2; Smad4; and gp130, which transduces signals from the pro-inflammatory cytokines IL-6 and IL-11 (reviewed in [7]). The PTEN/PI3K/Akt pathway, which is frequently deregulated in human gastric cancer, was also recently shown to promote development of mouse gastric cancer [8]. Many of these models have a protracted tumor latency, incomplete penetrance, and a low frequency of invasion and metastasis. In addition, little is known of the cell of origin of advanced gastric cancer.

Deregulated activation of the Hedgehog (Hh) pathway is an oncogenic driver for the common skin cancer, basal cell carcinoma (BCC), and a subset of medulloblastomas (reviewed in [9]). In both of these cancers, tumors arise due to uncontrolled activation of Hh signaling in progenitor cells whose proliferation is normally regulated by transient, physiologic Hh signaling [10]. Elevated Hh signaling activity has also been detected in a broad range of other cancers, but the role of the Hh pathway in the genesis of neoplasms other than BCC and medulloblastoma has not been well established [11]. Several reports have described alterations in the Hh pathway in gastric cancer (reviewed in [12]) and have linked alterations in the pathway to advanced tumor grade [13, 14], but animal models supporting the idea that uncontrolled Hh signaling can function as an oncogenic driver in stomach have not been reported.

Here we show that GLI2A, an activator form of the Hh pathway transcription factor GLI2, drives rapid development of gastric adenocarcinomas from Lgr5-expressing gastric stem cells. Invasive gastric cancers are detected as early as three weeks after GLI2A induction, express several markers detected in human gastric cancers, undergo epithelial-mesenchymal transition (EMT), upregulate multiple pro-inflammatory cytokines implicated in human gastric cancer development, and rapidly activate mTOR/S6 signaling. Treatment with rapamycin to block mTOR impairs growth of GLI2A-driven gastric cancers while triggering aberrant induction of mucous production by tumor cells. Our studies point to a stem cell origin for murine gastric adenocarcinomas and highlight the functional relevance of crosstalk between the Hh/Gli2 and mTOR pathways in cancer.

## RESULTS

### An inducible mouse model that drives GLI2A expression in Lgr5+ stem cells and progeny

**Figure 1:**
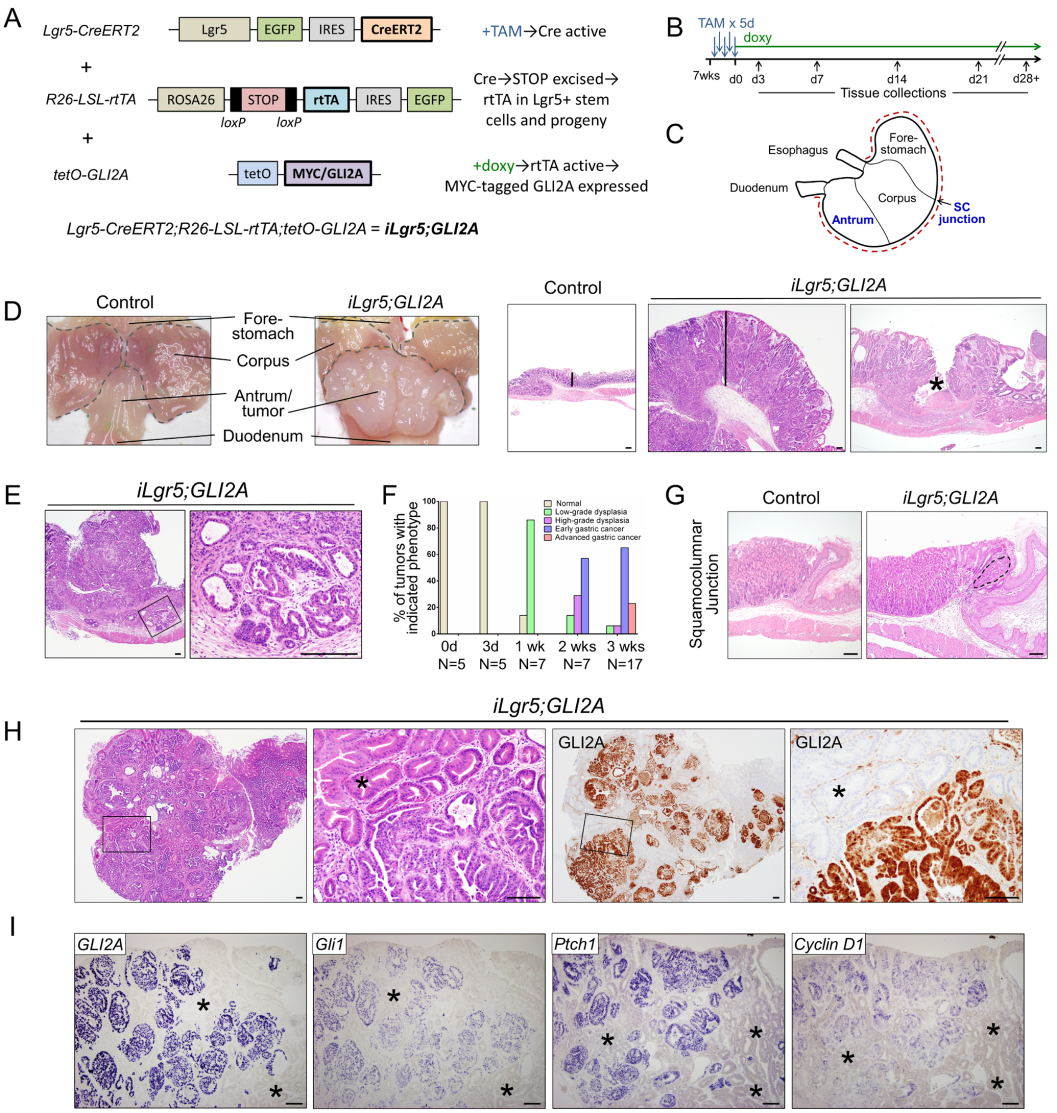
GLI2A expression in Lgr5+ stem cells drives rapid development of invasive gastric adenocarcinoma **A.** Triple-transgenic mouse model, which includes tamoxifen regulated *Lgr5-CreERT2* allele and doxycycline-regulated tet transactivator allele, to achieve tight, conditional GLI2A expression in adult *iLgr5;GLI2A* mice. **B.** General scheme for tamoxifen (TAM) dosing and doxy treatment. **C.** Stomach compartments and regions, with blue text indicating where the Lgr5 promoter is active. Red dashed line along greater curvature indicates where stomach was cut to expose mucosa (D) and prepare tissue for sectioning. **D.** Stomach harvested after 3 weeks of GLI2A induction contained large polypoid tumors in antrum that histologically resemble human gastric adenocarcinomas. Vertical lines in right panels illustrate marked thickening of tumor relative to control antral mucosa, and the asterisk indicates ulceration. **E.** Invasion of tumor cells into the submucosa with formation of atypical gland-like structures. **F.** Histologic scoring showing rapid neoplastic progression in *iLgr5;GLI2A* mice, with 88% of mice exhibiting either early or advanced gastric cancer at 3 weeks. **G.** Early tumor development (dashed line) near the squamocolumnar junction. **H.** Full-blown gastric tumors showed histological heterogeneity with two distinct epithelial morphologies: highly disorganized, atypical-appearing cells that express GLI2A, with neighboring GLI2A-negative hyperplastic antral glands (asterisk). **I.** RNA *in situ* hybridization detected canonical Hh target genes (*Gli1, Ptch1,* & *Cyclin D1*) in GLI2A-expressing, but not GLI2-negative (asterisks), tumor epithelium. Scale bars: 100 μm.

We used a triple-transgenic model engineered to express an activated GLI2 allele, *GLI2A*, in Lgr5-expressing stem cells and their progeny in adult mice when treated with doxycycline [15]. This model includes 1) a Cre-expressing driver, *Lgr5-EGFP-IRES-CreERT2* [16]; 2) a transgene carrying a Cre-inducible reverse tetracycline transactivator (rtTA) inserted into the broadly-expressed ROSA locus (*R26-LSL-rtTA-IRES-EGFP*) [17]; and 3) an effector transgene (*tetO-GLI2A*, also called *tetO-GLI2ΔN*) [15], which activates GLI2A expression in cells expressing rtTA upon treatment with doxycycline (Figure 1A). The Cre driver defines the cell population or lineage that initially expresses rtTA, and in the presence of doxy, GLI2A. Use of a CreER driver provides an additional level of control since the timing of Cre-mediated recombination and rtTA expression is controlled by the administration of tamoxifen (Figure 1A, 1B). Because rtTA is under the control of the ubiquitous ROSA promoter, its expression after recombination is sustained and not restricted to cells in which the Cre driver is active. Most of the data presented in this report are based on studies using triple-transgenic *Lgr5-EGFP-IRES-CreERT2;R26-LSL-rtTA-IRES-EGFP;tetO-GLI2A* mice, abbreviated *iLgr5;GLI2A*. In stomach of adult mice, the *Lgr5-CreERT2*-containing allele drives recombination in stem cells at the base of gastric antral glands and in the first gland of the corpus near the squamocolumnar junction (Figure 1C) [18].

### GLI2A expression in Lgr5+ stem cells drives rapid development of invasive gastric adenocarcinoma

Activation of GLI2A in Lgr5+ gastric stem cells led to the rapid development of gastric tumors which frequently replaced much of the normal antrum (Figure 1D). The antral tumor phenotype was highly penetrant, with 92% of *iLgr5;GLI2A* mice (*N* = 37) developing grossly visible tumors after 3 weeks of doxycycline treatment. H&E staining revealed large tumor masses with morphologic features similar to those seen in human gastric adenocarcinoma, including loss of differentiated cell types, tumor nodules containing multiple layers of disorganized epithelial cells, cytologic atypia, and abundant tumor stroma with a mixed inflammatory infiltrate (Figure 1D, 1E, Supplementary Figure 1, and below). Some tumors were ulcerated (Figure 1D); in addition, tumor cells sometimes invaded the submucosa and muscularis propria (Figure 1E, Supplementary Figure 1). Both of these findings are also seen in advanced gastric cancer in humans.

We analyzed tissue sections from a cohort of *iLgr5;GLI2A* mice (*N* = 41) euthanized at several time-points (Figure 1F) to gain additional insight into the process of neoplastic progression, with representative examples of histologic scoring shown in Supplementary Figure 1. One week after transgene induction, 86% of mice contained regions of low-grade dysplasia; by two weeks, 43% of mice had either low-grade or high-grade dysplasia, with the remaining 57% of mice scored as early gastric cancer; by three weeks, 65% of mice were scored as having early gastric cancer and 23% as advanced gastric cancer, with dysplasia noted in the remaining 12% (Figure 1F). Although grossly visible tumors in stomachs of *iLgr5;GLI2A* mice were limited to the gastric antrum, the area near the first gastric gland of the corpus at the squamocolumnar junction (Figure 1C) also frequently contained disorganized, dysplastic-appearing cells (Figure 1G), reflecting the expression pattern of the *Lgr5-CreER* driver in adult mice [18].

Human gastric adenocarcinomas frequently exhibit intratumor heterogeneity [19, 20], which was also detected in *iLgr5;GLI2A* mice. Full-blown tumors contained epithelial cells with two distinctive morphologies: disorganized cells frequently exhibiting cytologic atypia and a high nuclear to cytoplasmic ratio; and neighboring hyperplastic gastric glands composed of cells with abundant eosinophilic cytoplasm, an eccentric nucleus, and little or no atypia (Figure 1H). Expression of the GLI2A transgene, detected by immunostaining for the MYC epitope tag, was detected only in the disorganized/dysplastic tumor cells (Figure 1H). Elevated expression of Hh target genes, based on *in situ* hybridization, was detected in *GLI2A*-expressing tumor cells but not in neighboring hyperplastic gastric glands (Figure 1I). GLI2A was not detected in the few *iLgr5;GLI2A* mice that did not develop gastric lesions, establishing a strict correlation between transgene expression and tumorigenesis, and suggesting that stem cell-targeted GLI2A is sufficient to drive rapid gastric cancer development. GLI2A thus drives cell-autonomous activation of Hh signaling in transgene-expressing gastric tumor cells, and this is associated with robust hyperplasia of neighboring gastric epithelia.

### Lgr5-driven GLI2A expression fails to drive epithelial tumor development in intestine

The Lgr5 promoter also targets epithelial stem cells in skin and intestine, and while we previously showed that expression of GLI2A in this cell population in skin gives rise to basal cell carcinomas [15], we did not detect grossly apparent tumors in intestines of *iLgr5;GLI2A* mice. Histology of tissue collected at a time when GLI2A expression yielded massive gastric tumors confirmed the absence of epithelial tumors in intestine, although the tips of some villi contained tumor-like collections of mesenchymal cells (Supplementary Figure 2A). In contrast to findings in the gastric antrum, GLI2A-expressing epithelial cells were not detected by immunostaining in intestine of *iLgr5;GLI2A* mice 3-4 weeks after transgene activation (Supplementary Figure 2B). To ascertain whether GLI2A-expressing cells were present at earlier time points, we performed additional MYC immunostaining. GLI2A-expressing epithelial cells were detected in intestinal crypts and the base of villi at day 1 and 3 but were markedly less abundant 7 days after transgene induction, and undetectable at 3 weeks; in contrast, GLI2A expression was robust in mesenchymal aggregates (Supplementary Figure 2B). Some GLI2A-expressing epithelial cells co-expressed cleaved caspase 3 (Supplementary Figure 2C), suggesting that elimination via apoptosis contributes to the loss of GLI2A-expressing cells and lack of epithelial tumor development in intestine of *iLgr5;GLI2A* mice.

### Proliferation and lineage marker expression in GLI2A-driven gastric adenocarcinomas

**Figure 2:**
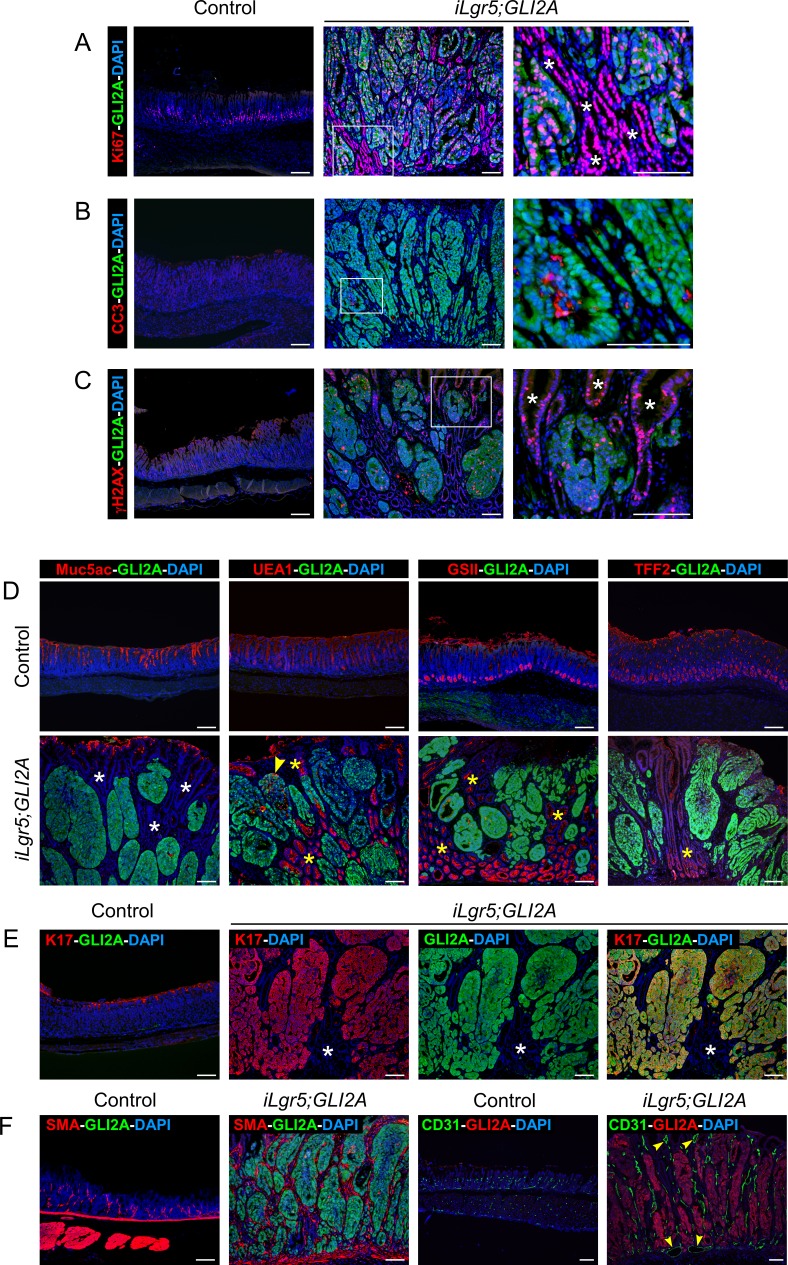
Proliferation and lineage marker expression in GLI2A-driven gastric adenocarcinomas **A.** Double-immunostaining for Ki67 and GLI2A revealed widespread proliferation in GLI2A-expressing gastric tumor cells as well as neighboring GLI2A-negative gastric epithelial cells (white asterisks). **B.** Increased apoptosis, based on immunostaining for cleaved caspase 3 (CC3), was detected in a subset of GLI2A-expressing gastric tumor cells. **C.** DNA damage was detected both in GLI2A-expressing tumor cells and GLI2A-negative hyperplastic gastric epithelium (white asterisks) by immunostaining for γH2AX. **D.** Expression of gastric mucins was detected infrequently (arrowhead) in GLI2A-expressing tumor cells, whereas hyperplastic GLI2A-negative gastric epithelium frequently expressed Tff2 and mucins detected by lectins UEA1 and GSII (yellow asterisks), but not Muc5ac (white asterisks). **E.** Co-localization of K17 and GLI2A in nearly all gastric tumor cells. Asterisks show GLI2-negative epithelia which do not express K17. **F.** Immunostaining for either SMA or CD31 revealed an increased number of myofibroblasts and blood vessels, respectively. Large vessels indicated with arrowheads. Scale bars: 100 μm.

GLI2A-expressing gastric tumors were highly proliferative, based on immunostaining for Ki67. Co-immunostaining revealed that both the GLI2A-expressing tumor cells and neighboring GLI2A-negative gastric epithelium were highly proliferative (Figure 2A). The rate of cell death was increased in tumor cells when compared to normal antral cells based on immunostaining to detect cleaved caspase 3 (CC3) (Figure 2B). γH2AX, a DNA damage marker frequently up-regulated in cancer [21], was detected in both GLI2A-expressing tumor cells and GLI2A-negative hyperplastic gastric epithelium (Figure 2C).

Additional staining of full-blown tumors for markers expressed in normal antral glands revealed that GLI2A-expressing tumor cells did not express Muc5ac or Tff2, and infrequently stained with the mucin-binding lectins GSII and UEA1 (Figure 2D). Each of these was detected in control antrum and, with the exception of Muc5ac, were also highly expressed in some of the hyperplastic GLI2A-negative gastric epithelia within tumor masses (Figure 2D, yellow asterisks). Keratin 17 was detected in nearly all GLI2A-expressing tumor cells (Figure 2E). K17 is also induced in human gastric cancers, with increased expression levels associated with a poor prognosis [22]. In addition to changes involving epithelial tumor cells, tumor stroma contained an increased number of SMA-expressing myofibroblasts and was highly vascularized, based on CD31 immunostaining (Figure 2F).

### EMT in GLI2A-driven gastric cancers is associated with canonical Wnt/β-catenin signaling

**Figure 3:**
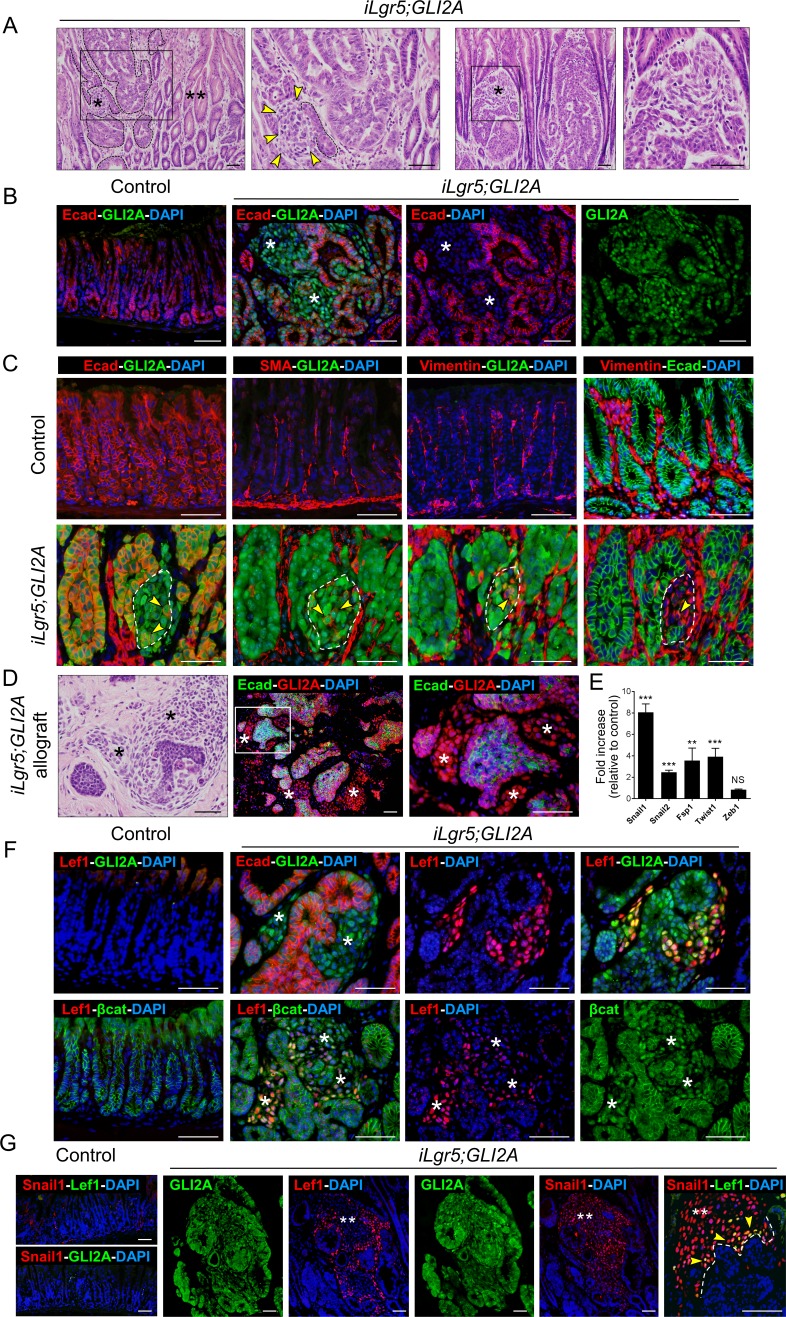
EMT is associated with canonical Wnt/β-catenin signaling and Snail1 expression **A.** Histology of gastric tumors in two left panels shows dysplastic epithelial cells (dashed lines), contiguous mesenchymal-appearing cells (single asterisk in low mag, arrowheads in high mag images), and hyperplastic epithelium (double asterisks). Two right panels show another representative tumor with mesenchymal-appearing region (asterisk). **B.** Reduced or undetectable expression of the epithelial marker E-cadherin (Ecad) in GLI2A-expressing tumors cells in regions with presumed EMT (asterisks). **C.** Lower left panel shows loss of Ecad expression in region of early EMT (dashes lines), with only residual staining in a few cells (arrowheads). Neighboring sections show appearance of SMA and vimentin with occasional co-expression of Ecad and vimentin (arrowheads). **D.** Mesenchymal-appearing cells (asterisks) in tumor allografts grown in nude mice. Ecad is lost in GLI2A-expressing mesenchymal cells. **E.** Transcripts encoding EMT markers were measured by qRT-PCR in RNA isolated from control antra (*N* = 8) and *iLgr5;GLI2A* gastric tumors (*N* = 8). Data are expressed as means +/− SEM (**P = 0.0019 and ***P = 0.0002). **F.** Canonical Wnt signaling restricted to regions of EMT, based on immunostaining showing Lef1 and nuclear β-catenin restricted to non-epithelial Ecad-/GLI2A+ tumor cells (asterisks). **G.** Immunostaining for Lef1 and the EMT marker Snail1 in larger regions of EMT revealed coexpression in cells at the periphery (arrowheads) adjacent to GLI2A+ epithelial tumor cells (dashed line). Snail1 expression was also detected more centrally in mesenchymal GLI2A+ tumor cells that no longer expressed detectable levels of Lef1 (white double asterisks). Scale bars: 50 μm.

In addition to the epithelial cells which comprised the bulk of gastric tumors in *iLgr5;GLI2A* mice we also detected variable numbers of GLI2A-expressing mesenchymal-appearing cells that were occasionally contiguous with tumor epithelium (Figure 3A, single asterisks), highly suggestive of cells that had undergone epithelial-mesenchymal transition (EMT). GLI2A-expressing cells in these regions had reduced or undetectable levels of the epithelial marker E-cadherin (Ecad), expressed SMA and vimentin, and rarely, co-expressed Ecad and vimentin (Figure 3B, 3C). Mesenchymal GLI2A-expressing cells were seen in nearly all primary tumors and in allografts of *iLgr5;GLI2A* gastric tumors which grew subcutaneously in nude mice (Figure 3D). Real-time PCR on samples from *iLgr5;GLI2A* gastric tumors revealed upregulation of mRNA encoding multiple EMT markers, including *Snail1* (8.1-fold), *Snail2/Slug* (2.5-fold), *FSP1* (3.6-fold), and *Twist1* (3.9-fold) (Figure 3E).

Canonical Wnt signaling is activated in certain Hh pathway-driven tumors [23, 24], and has been implicated in the development of a subset of gastric cancers and in EMT. We therefore performed immunostaining of gastric tumors from *iLgr5;GLI2A* mice to assess Lef1 expression and nuclear translocation of β-catenin, markers of canonical Wnt signaling. Lef1 and nuclear β-catenin were limited to Ecad-deficient/GLI2A-expressing mesenchymal-appearing tumor cells (Figure 3F), establishing a strict relationship in these tumors between canonical Wnt signaling activity and EMT. In tumors containing larger regions of EMT, Lef1 was detected adjacent to epithelial tumor cells (Figure 3F, 3G), suggesting activation of Wnt signaling early, and transiently, during EMT. The EMT marker Snail1 was also expressed in EMT transition zones but persisted in mesenchymal cell populations that no longer expressed Lef1 (Figure 3G, double asterisks). GLI2A+/Ecad+ gastric epithelial tumor cells also occasionally expressed Snail1 although generally at levels lower than in GLI2A+/Ecad− cells (not shown). Collectively, these data establish that EMT in GLI2A-expressing gastric adenocarcinomas is tightly linked to transient activation of canonical Wnt/β-catenin signaling and sustained expression of Snail1 and other EMT markers.

### Inflammation and signaling alterations in GLI2A-driven gastric adenocarcinomas

**Figure 4:**
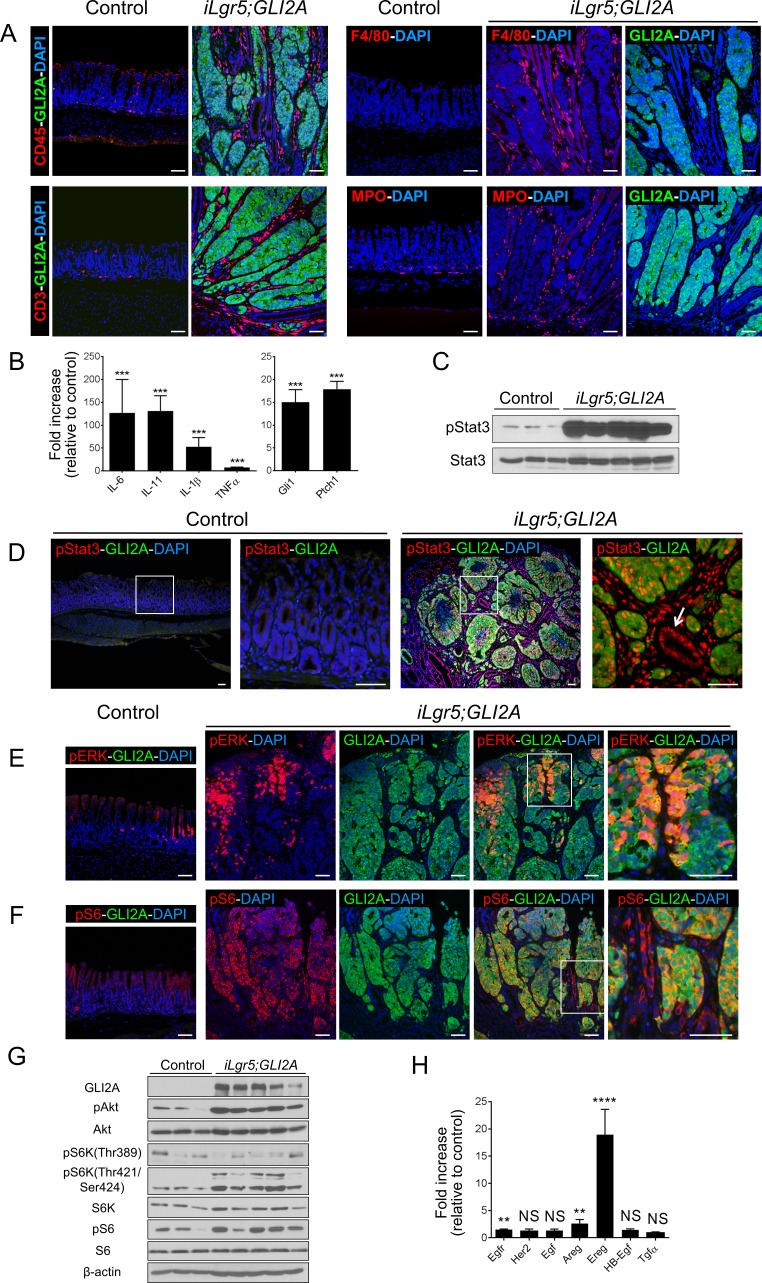
Inflammation and signaling alterations in GLI2A-driven gastric adenocarcinomas **A.** Increased number of inflammatory cells in GLI2A-expressing gastric tumors detected by immunostaining for CD45 (total myeloid cells), CD3 (T cells), F4/80 (macrophages), and myeloperoxidase (MPO; neutrophils). F4/80 and MPO immunostaining were done in adjacent sections relative to GLI2A immunostaining. **B.** Upregulation of Hh target genes (*Gli1,Ptch1*) and transcripts encoding multiple pro-inflammatory cytokines was assessed by qRT-PCR in RNA isolated from control mice (*N* = 8) and *iLgr5;GLI2A* gastric tumors (*N* = 8). Data are expressed as means +/− SEM (***P = 0.0002). **C.** Immunoblotting using lysates from antral tumors and control antrum shows upregulation of the signaling effector pStat3. **D.** Negligible levels of pStat3 were detected in control antrum (left panels), whereas in tumors, pStat3 was detected in GLI2A-expressing tumor cells, stromal cells, and GLI2A-negative hyperplastic epithelia (arrow). **E.** pERK and GLI2A were co-expressed in a variable proportion of cells typically located in the uppermost portion of tumors. **F.** Phosphorylation/activation of the mTOR target S6 was detected by pS6 immunostaining, which was uniformly elevated in tumor cells and, at lower levels, in hyperplastic, GLI2A-negative gastric epithelia. **G.** Immunoblot analysis showing upregulation of mTOR signaling in GLI2A tumors, with increased levels of pAkt, pS6K, and pS6. H) Transcripts for Egfr, Her2, and multiple Egfr ligands were measured by qRT-PCR in RNA isolated from control antra (*N* = 8) and *iLgr5;GLI2A* gastric tumors (*N* = 8). Data are expressed as means +/− SEM [****P < 0.0001 (*Ereg*), **P = 0.0037 (*Egfr*), and **P = 0.0079 (*Areg*)]. Scale bars: 50 μm.

Given the central role of inflammation in the development of gastric cancer in humans and experimental rodent models [7], we examined the inflammatory infiltrate in GLI2A-expressing gastric cancers by immunostaining. The numbers of CD3 (T cells), CD45 (total myeloid cells), F4/80 (macrophages), and myeloperoxidase (MPO) expressing cells (neutrophils) were markedly increased in gastric tumors 3 weeks after GLI2A induction (Figure 4A). Expression of transcripts encoding multiple pro-inflammatory cytokines previously implicated in gastric cancer was also elevated, including IL-6, IL-11, IL-1β, and TNFα (Figure 4B). In addition, Stat3, a pivotal transcriptional effector mediating cytokine responses contributing to several inflammation-associated cancers (reviewed in [25, 26]), was activated/phosphorylated diffusely in gastric adenocarcinomas in both epithelium as well as stroma, with negligible pStat3 immunostaining in control antrum (Figure 4C, 4D).

In addition to cytokine-driven activation of pStat3 signaling, human gastric cancers exhibit alterations in several other signaling pathways, including receptor tyrosine kinase/MAPK and PI3K/Akt/mTOR pathways (reviewed in [27]). Immunostaining revealed MAPK activation, based on pERK immunostaining, in a variable proportion of cells that were typically located in the uppermost portion of tumors (Figure 4E). This pattern resembles findings in another mouse model of gastric cancer [28] and suggests that pERK signaling is not a major contributor to proliferation in most GLI2A-expressing tumor cells. In contrast to pERK, immunostaining to detect phosphorylated ribosomal protein S6 (pS6), an indicator of mTOR signaling, was detected broadly throughout the tumor masses. pS6 expression was uniformly elevated in GLI2A-expressing tumor cells and was also detected at lower levels in most of the hyperplastic, GLI2A-negative gastric glands and stromal cells (Figure 4F). Immunoblot analysis confirmed upregulation of mTOR signaling in GLI2A tumors, likely via the mTORC1 complex, with increased activation (phosphorylation) of the upstream effector Akt and downstream targets S6K and S6 (Figure 4G). Given the proposed involvement of EGFR and HER2 signaling in some gastric cancers [29] and previously-reported crosstalk between the Hh and EGFR pathways [30, 31], we determined the expression levels of Egfr, Her2, and Egfr ligand mRNAs by real-time PCR. Egfr, Amphiregulin (Areg), and Epiregulin (Ereg) transcripts were upregulated 1.5-, 2.6-, and 19-fold, respectively, in gastric tumors arising in *iLgr5;GLI2A* mice (Figure 4H).

### Induction of proliferation and S6 phosphorylation at early stages of gastric tumor development

**Figure 5:**
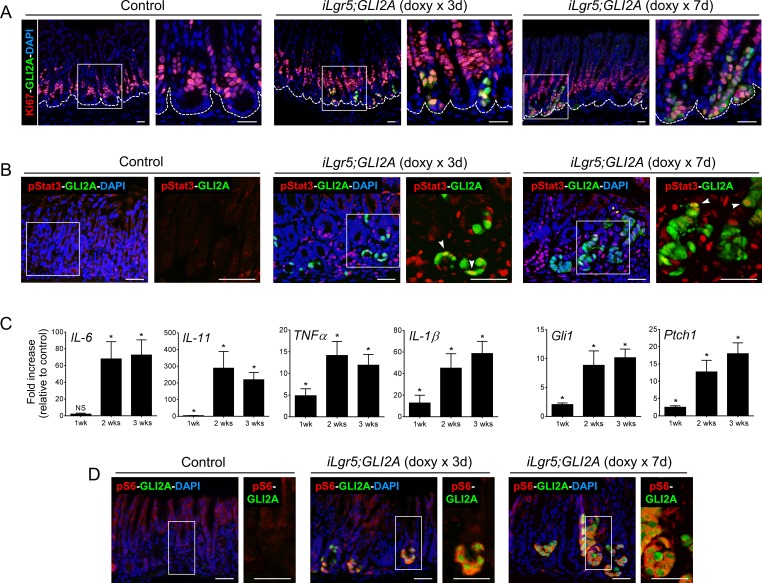
Induction of proliferation and S6 phosphorylation are detected at early stages of tumor development **A.** Double-immunostaining revealed Ki67+/GLI2A+ cells in the largely quiescent base of gastric antral glands (left panels) at 3 days and 7 days after starting doxy treatment in *iLgr5;GLI2A* mice. **B.** Double-immunostaining for pStat3 and GLI2A revealed few GLI2A+ cells co-expressing pStat3 (white arrowheads) in the base of antral glands at 3 days and 7 days after starting doxy treatment in *iLgr5;GLI2A* mice. **C.** Temporal activation pattern of transcripts for multiple pro-inflammatory cytokines and Hh target genes (*Gli1,Ptch1*), measured by qRT-PCR in RNA isolated from controls (*N* = 5 for each time point) and *iLgr5;GLI2A* gastric tumors (*N* = 4 for each time point) at 1, 2, and 3 weeks post doxy-induction. Data are expressed as means +/− SEM (*P = 0.0159). **D.** Double-immunostaining revealed a strict correlation between expression of GLI2A and pS6 in the base of gastric glands during early stages of tumor development. Scale bars: 20 μm.

We collected tissue at 3 and 7 days after transgene activation to examine cytokine/Stat3 and mTOR/S6 signaling during tumor initiation and early expansion. In control mice, Ki67+ cells are rarely detected in the stem cell compartment at the base of antral glands but are abundant in transit-amplifying cells of the proliferative zone located several cell diameters above (Figure 5A). In contrast, 3 days after starting doxy treatment in *iLgr5;GLI2A* mice, GLI2A+ cells in the base of antral glands co-expressed Ki67, and by 7 days, columns of GLI2A+/Ki67+ cells extended from the base into the body of antral glands (Figure 5A). These data suggest that activation of GLI2A in Lgr5+ stem cells residing in the relatively quiescent base of antral glands is sufficient to stimulate proliferation and upward expansion of nascent tumor cells during early stages of tumor development.

We next examined the levels of pSTAT3, pS6, and pro-inflammatory cytokines at early stages of GLI2A-driven tumorigenesis. In contrast to the widespread and nearly uniform activation of STAT3 seen in full-blown tumors, analysis at 3 and 7 days after doxy treatment revealed small foci of cells expressing pStat3 and only a subset of these cells co-expressed GLI2A, indicating that GLI2A does not activate Stat3 in a cell autonomous manner (Figure 5B). Instead, infiltration of inflammatory cells during tumor expansion likely creates a cytokine-rich microenvironment leading to STAT3 activation in responsive cell populations. In keeping with this concept, transcripts encoding IL-6 and IL-11, key cytokines involved in inflammation-associated Stat3 activation in cancer [32-34], were marginally elevated after one week of GLI2A expression but highly induced by 2 and 3 weeks (Figure 5C), when Stat3 activation is widespread (Figure 4C, 4D). In contrast to the delayed activation of pro-inflammatory cytokine signaling and Stat3 activation, immunostaining at early time-points after doxy administration revealed a strict correlation between expression of GLI2A and pS6 at the base of gastric glands. Essentially all GLI2A-expressing cells co-stained for pS6, which was not detected in surrounding transgene-negative epithelial cells (Figure 5D), suggesting that GLI2A stimulates mTOR/S6K/S6 signaling cell-autonomously during tumor initiation and early progression.

### Growth of GLI2A-driven gastric cancers is dependent on mTOR/S6 signaling

**Figure 6:**
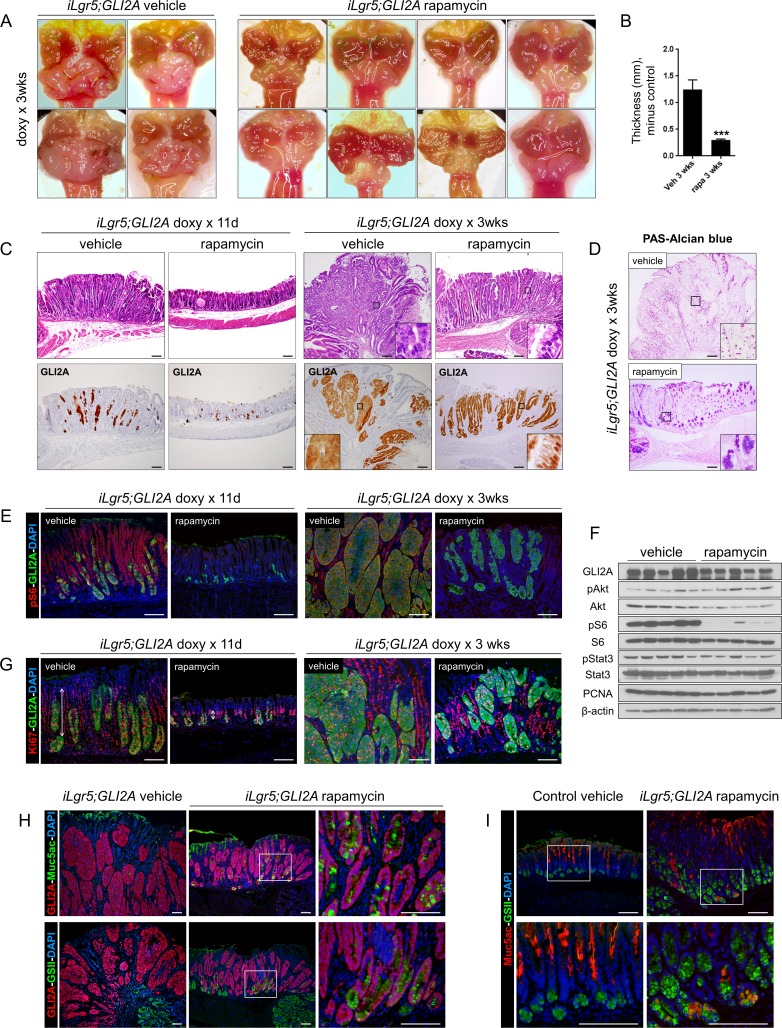
mTOR signaling contributes to growth of GLI2A-driven gastric cancers **A.** Gross appearance of stomachs from *iLgr5;GLI2A* mice showing impaired growth of tumors in rapamycin-treated mice when compared to vehicle-treated. **B.** Decreased antral tumor thickness in rapamycin-treated (*N* = 8) versus vehicle-treated (*N* = 6) *iLgr5;GLI2A* mice. Data are expressed as means +/− SEM (***P = 0.0007). **C.** Markedly reduced mass of GLI2A-expressing tumor cells as well as GLI2A-negative hyperplastic epithelia in rapamycin-treated *iLgr5;GLI2A* mice. Insets in right panels show appearance of cells with abundant pale-staining cytoplasm reminiscent of mucin, seen only in tumors from rapamycin-treated mice. **D.** PAS-Alcian blue staining detects cells containing abundant mucin only in tumor cells from rapamycin-treated mice. **E.** Immunostaining for the mTOR pathway reporter pS6 is lost in GLI2A-expressing tumor cells as well as hyperplastic gastric epithelia of rapamycin-treated mice, confirming effective blockade of mTOR signaling. **F.** Immunoblot analysis of tumor lysates from rapamycin-treated mice corroborates pS6 immunostaining results in E), showing nearly complete inhibition of pS6 expression relative to tumors from vehicle-treated mice. In contrast, treatment with rapamycin had minimal effects on pAkt or pStat3 expression. **G.** Immunostaining for Ki67 and GLI2A in tumors from rapamycin-treated mice revealed a striking reduction in the proliferative compartment of GLI2A-negative epithelial cells, particularly at 11 days post-induction (vertical arrows in left panels). **H.** In contrast to the undifferentiated vehicle-treated tumors in *iLgr5;GLI2A* mice, tumor cells from rapamycin-treated mice expressed Muc5ac and GSII-binding mucins. **I.** Some tumor cells from rapamycin-treated mice aberrantly expressed Muc5ac and bound GSII lectin in the same cell. Scale bars: 100 μm.

Given the early and sustained induction of pS6 in GLI2A-expressing gastric cancers, we tested the function of mTOR signaling in tumorigenesis by treating mice with the mTOR inhibitor rapamycin, which resulted in a strong inhibition of tumor growth in *iLgr5;GLI2A* mice (Figure 6A). Histology of rapamycin-treated tumors collected after 11 days or 3 weeks revealed substantially fewer GLI2A-expressing tumor cells at both time points and a markedly lower tumor thickness at 3 weeks (Figure 6B, 6C). Moreover, some GLI2A-expressing tumor cells in rapamycin-treated mice had an altered morphology, with abundant cytoplasm and eccentric nucleus suggestive of differentiating, mucous-producing cells (Figure 6C, insets). In keeping with this possibility, a subset of tumor cells from rapamycin-treated mice, but not vehicle-treated mice, stained with PAS/Alcian blue, a histochemical stain for mucins (Figure 6D).

The efficacy of rapamycin at blocking mTOR signaling, specifically mTORC1, was confirmed by immunostaining and immunoblotting for pS6, which was markedly reduced in tumors from rapamycin-treated versus vehicle-treated mice (Figure 6E, 6F). Activation of pAkt in tumors (Figure 4G), which lies upstream of mTORC1 but downstream of mTORC2, was not reduced by rapamycin treatment (Figure 6F), arguing that the main mTOR complex involved in GLI2A-mediated signaling crosstalk is mTORC1.

In early-stage tumors collected 11 days after transgene induction, rapamycin markedly inhibited expansion of the proliferative compartment of GLI2A-negative epithelial cells (Figure 6G, vertical lines); this difference was no longer apparent at 3 weeks. The rate of apoptosis seen in control tumors (see also Figure 2B) was reduced in mice treated with rapamycin for 3 weeks (Supplementary Figure 3), arguing that increased cell death at this time point was not contributing to the smaller tumor size.

Interestingly, strong expression of Muc5ac and binding of GSII lectin were detected at 3 weeks in GLI2A-expressing tumor cells from rapamycin but not vehicle-treated mice (Figure 6H), in keeping with H&E and PAS/Alcian blue staining (Figure 6C, 6D). Different cell populations in normal stomach express Muc5ac and bind GSII lectin but these markers were co-localized in cells from rapamycin-treated mice (Figure 6I), suggesting that some tumor cells activate an aberrant differentiation program when mTOR signaling is inhibited. Finally, pStat3 was still broadly expressed in rapamycin-treated tumors (Figure 6F, Supplementary Figure 4), suggesting that mTOR signaling is not likely to be a major contributor to pStat3 activation in this model. Taken together, these data show that the early growth phase of GLI2A-expressing gastric cancers, but not tumor initiation, is dependent on mTOR/S6 signaling, which may promote tumor growth partly by maintaining cells in an undifferentiated, proliferative state.

## DISCUSSION

Gastric adenocarcinoma remains one of the major causes of worldwide cancer mortality but major gaps exist in our understanding of the biology and molecular basis of this lethal malignancy. Here we show that tumors with multiple features mimicking invasive human gastric adenocarcinoma develop within 3 weeks after expressing the Hh pathway transcription factor GLI2A in mouse stomach. Tumors arise from Lgr5-expressing stem cells in the gastric antrum, undergo rapid malignant progression, exhibit foci of EMT, and activate multiple pathways previously linked to human gastric cancer. These data suggest that at least some forms of gastric cancer may arise from Lgr5-expressing stem cells; identify *iLgr5;GLI2A* mice as a novel model of invasive gastric adenocarcinoma; support the notion that deregulated Hh/GLI signaling may contribute to human gastric tumorigenesis; and point to an important role for deregulated mTOR signaling, likely via the mTORC1 complex, acting downstream of GLI2A in this model.

GLI2A-driven gastric cancers exhibited striking intratumor heterogeneity. Immunstaining for the MYC-tagged GLI2A transgene product identified dysplastic GLI2A-expressing tumor cells adjacent to highly-proliferative GLI2A-negative gastric epithelial cells, and *in situ* hybridization of full-blown tumors revealed Hh target gene expression only in transgene-expressing cells (Figure 1). Although hyperplasia of normal epithelia adjacent to gastrointestinal tumors is generally not discussed in the context of tumor heterogeneity, this is a common finding in human gastric cancers (J.K.G., unpublished observations) and may be a sizeable contributor to overall tumor mass. One proposed mechanism for this phenomenon is paracrine activation of EGFR on non-transformed epithelia via tumor-derived EGFR ligands [35], and the elevated expression of Areg and Ereg transcripts in GLI2A-expressing tumors (Figure 4) is in keeping with this possibility. Other potential growth-promoting signals are the abundant pro-inflammatory cytokines present in this model (Figures 4, 5), which have been previously linked to gastric cancer development in humans and are reflected in the widespread activation of STAT3 in tumor epithelia. Additional studies will be required to identify the paracrine acting factor(s) driving the robust hyperplasia of normal gastric epithelial cells within the tumor microenvironment.

Another example of intratumor heterogeneity is EMT, a prominent feature of gastric adenocarcinomas arising in *iLgr5;GLI2A* mice (Figure 3). Although Hh signaling has been linked to EMT in previous studies (reviewed in [36]), EMT was detected only in GLI2A+ regions of gastric tumors that also exhibited canonical Wnt/β-catenin signaling and Snail1 expression (Figure 3). EMT in this model appears to be a stable phenotype since mesenchymal GLI2A+ tumor cells are common in nude mouse allografts derived from gastric cancers that had arisen in *iLgr5;GLI2A* mice. While we cannot formally rule out the possibility that mesenchymal-appearing tumor cells in this model reflect the outgrowth of a small number of Lgr5+ mesenchymal cells or expansion of a bone marrow-derived cell population, the contiguous appearance of ECad+ and ECad− tumor cells, and the occasional colocalization of epithelial and mesenchymal markers in tumor cells, argue against this possibility.

The rapid appearance and progression of gastric tumors in GLI2A-expressing mice contrasts with apparently less aggressive gastric tumors arising in several other genetically-engineered mouse models (reviewed in [7]). Thus, a substantial proportion of gastric tumors from *iLgr5;GLI2A* mice were locally invasive, with dysplastic islands of tumor cells extending into the submucosa, or deeper, into the muscularis propria. Although metastases were not detected in the *iLgr5;GLI2A* mice that we examined, these mice typically had to be euthanized by 4 weeks after transgene induction due to the severity of skin tumors, and this may not have been sufficient time for dissemination and expansion of gastric tumor cells in lymph nodes or distant sites. Regardless, our success at growing multiple (4/5) gastric tumors subcutaneously in nude mice supports the assertion that tumors arising in this model are malignant.

Multiple reports have linked alterations in the Hh pathway to gastric cancer (reviewed in [12]), but convincing evidence showing that deregulated Hh signaling can function as an oncogenic driver in an *in vivo* model of gastric cancer has not been presented. The Hh pathway is active in normal gastric mucosa, with epithelium-derived Hh ligands signaling in a strictly paracrine fashion to mesenchymal cells that express Hh signaling effectors and target genes (*Gli1, Gli2, Ptch1*). Our data show that aberrant activation of GLI2 transcriptional activity in Lgr5+ antral stem cells and their progeny is sufficient for gastric tumorigenesis in mice and support the idea that Hh/GLI2 signaling could also contribute to gastric tumorigenesis in humans. A recent genome sequencing study provides data in keeping with this possibility in a subset of gastric cancers. GLI3 was mutated in 6.9% of microsatellite-stable gastric cancers [4]: because GLI3 generally functions as a transcriptional repressor (reviewed in [37]), its loss would be expected to lead to a net increase in Gli transcriptional activity.

In addition to its expression in gastric antral stem cells, the Lgr5 promoter is also active in hair follicle and intestinal stem cells, but in contrast to skin and stomach, GLI2A fails to generate epithelial tumors in the intestine (Supplementary Figure 2). Analysis at early time-points after transgene induction argues that this is not due to a tissue-specific deficiency in GLI2A protein expression or accumulation. Instead, Lgr5-targeted, GLI2A-expressing epithelial cells in intestine appear to be eliminated through apoptosis prior to tumor development. The lack of GLI2A-driven epithelial tumors in intestine is in stark contrast to widespread intestinal adenoma development following activation of oncogenic Wnt/β-catenin signaling using the same *Lgr5-CreER* strain [38, 39]. These data underscore the importance of tissue context in defining responsiveness of Lgr5-targeted epithelial stem cells to deregulated Hh signaling: Lgr5+ gastric stem cells readily form tumors in response to GLI2A, while Lgr5+ intestinal stem cells appear to be resistant.

Tumor cells in *iLgr5;GLI2A* mice activate multiple signaling pathways previously shown to be deregulated in human gastric cancer (Figure 4) [40]. Most strikingly, 3 days after transgene induction mTOR/S6 signaling was activated strictly in GLI2A-expressing nascent tumor cells at the base of gastric glands (Figure 5), establishing a tight association between the Hh/Gli2 and mTOR pathways during tumor initiation. During tumor expansion, mTOR/S6 signaling persisted in GLI2A+ tumor cells and was also detected in GLI2A-negative epithelial cells, suggesting activation via a paracrine mechanism in the latter cell population. These findings are in keeping with studies implicating mTOR signaling in human gastric cancers (reviewed in [41]), mouse models of gastric cancer [8, 28], and a recent report identifying activating mutations in the upstream effector PIK3CA in a subset of human gastric cancers [6].

Blockade of mTOR signaling with rapamycin impaired GLI2A-driven gastric tumor growth and affected both proliferation and differentiation. At early stages the effects on cell proliferation were predominant: tumors from rapamycin-treated mice had fewer GLI2A+ tumor cells and a striking absence of hyperproliferative GLI2A-negative epithelial cells (Figure 6), suggesting that both cell-autonomous effects of GLI2A in tumor cells and paracrine signals driving proliferation in neighboring gastric epithelium are dependent on mTOR signaling. At later stages, tumors in rapamycin-treated mice remained smaller than in vehicle-treated mice, and numerous mucin-containing cells were detected within GLI2A+ tumor masses (Figure 6). Together, these data suggest that in addition to its requirement for hyperplasia of GLI2A-negative gastric glands in early tumors, mTOR signaling activity at later stages maintains the undifferentiated phenotype of GLI2A-expressing tumor cells, and both of these functions likely contribute to the impaired growth of tumors in rapamycin-treated mice. These results are reminiscent of studies in Wnt pathway-driven intestinal adenomas, where mTOR blockade also has anti-tumor activity linked to reduced tumor cell proliferation and increased differentiation [42, 43].

In summary, our data show that deregulated Hh/Gli2 signaling in Lgr5+ stem cells is sufficient to drive invasive gastric adenocarcinoma development in mice, point to a potential role for Hh/Gli signaling in a subset of human gastric cancers, underscore the functional significance of crosstalk between the Hh/Gli2 and mTOR signaling pathways in cancer, and highlight the divergent responsiveness of gastric versus intestinal Lgr5+ epithelial stem cells to oncogenic Hh signaling.

## MATERIALS AND METHODS

### Mouse strains, genotyping, transgene induction, and rapamycin treatment

To target transgene expression to Lgr5+ stem cells, we generated triple-transgenic mice, abbreviated *iLgr5;GLI2A*, by combining the following alleles: *Lgr5-EGFP-IRES-CreERT2* (JAX stock # 008875), *ROSA-LSL-rtTA-IRES-EGFP* (JAX stock # 005572), and *tetO-GLI2ΔN^380^* [15], referred to here as *tetO-GLI2A* (Figure 1A). Genotyping was performed with genomic DNA isolated from tail snips using the following primers and PCR parameters. To detect *Lgr5-EGFP-IRES-CreERT2*, 5′-CATGCTTCATCGTCGGTCC-3′ (forward) and 5′-GATCATCAGCTACACCAGAG-3′ (reverse), 412 bp product. To detect *ROSA-LSL-rtTA-IRES-EGFP*, 5′-CTAAGTCATCGCAATGGAGC-3′ (forward) and 5′-CCAGATCGAAATCGTCTAGC-3′ (reverse), 580 bp product. To detect *tetO-GLI2A*, 5′-CATGCTTCATCGTCGGTCC-3′ (forward) and 5′-GATCATCAGCTACACCAGAG-3′ (reverse), 522 bp product. Cycling parameters are as follows: 95°C for 5 minutes, [95°C for 30 seconds, 58°C for 30 seconds, 72°C for 30 seconds] for 35 cycles, and 72°C for 2 minutes.

To stimulate Cre activity and induce rtTA expresssion, we treated 7-8 week-old mice with tamoxifen (20 mg/ml in corn oil; Sigma-Aldrich) at 0.1 mg/g body weight, administered daily by oral gavage for 5 days. We then fed mice doxycycline-containing chow (1 g/kg; Bio-Serv) to induce rtTA-driven expression of GLI2A and animals were euthanized for tissue collection at the indicated times. For the first 3 days of doxycycline induction, mice also received 200 μg/ml doxycycline in drinking water with 5% sucrose (Figure 1B). For mTOR inhibition studies, tamoxifen-treated mice were treated for 3 days with daily intraperitoneal injections of 5 mg/kg rapamycin or vehicle (5% Tween 80 and 5% polyethylene glycol 400 in saline), GLI2A was induced with doxycycline, and rapamycin administration was continued every other day for the duration of the experiment. Use and care of animals and experimental protocols were approved by the University Committee on the Use and Care of Animals (Animal Use Protocol PRO00004440).

### Tissue collection, immunostaining, and *in situ* hybridization

Each stomach was cut along the greater curvature (Figure 1C), briefly rinsed, pinned in place serosal side down on filter paper (0.45 μm mixed cellulose esters filters, cat# HAWP02500, Millipore), and fixed in 4% paraformaldehyde (PFA) at 4°C overnight. Samples were transferred to 70% ethanol prior to processing and paraffin-embedding. For frozen sections, tissues were fixed in 4% PFA at 4°C for 1 hour, transferred to 30% sucrose/PBS overnight, embedded in Tissue-Tek OCT Compound (Sakura), and stored at −80°C. Immunostaining was performed as previously described [23] according to manufacturer's instructions, with minor modifications, using either VECTASTAIN ABC kit (Vector Laboratories, PK-4001) or M.O.M. kit (Vector Laboratories, PK-2200). For co-immunostaining, fluorescent secondary antibodies conjugated with DyLight 488 or DyLight 549 (Jackson ImmunoResearch) were used, and ProLong Gold Antifade Reagent with DAPI (Life Technologies #P36931) was used for mounting. Primary antibodies used for immunostaining are listed in Supplementary Table 1. Mucins were also detected using fluorochrome-conjugated lectins: *Ulex Europaeus* Agglutinin I (UEA I) (#B-1065; Vector Laboratories); and fluorescein-conjugated GSII lectin (Vector Laboratories). *In situ* hybridization was performed on paraffin-embedded sections. Riboprobe sequences, probe preparation, and hybridization protocols have been described previously [44].

### Immunoblot analysis

Freshly harvested stomachs were homogenized in ice-cold radio-immunoprecipitation assay lysis buffer containing phosphatase and protease inhibitor cocktail (Roche Applied Science). Protein lysates were cleared by centrifugation and stored at −80°C. Fifty μg of protein per lane was separated by SDS-polyacrylamide gel electrophoresis. Immunoblotting and detection were performed as previously described [45], with minor modifications. Antibodies used for immunoblotting are listed in Supplementary Table 2.

### Measurement of antral tumor thickness in rapamycin-treated mice

Average tumor thickness was determined from H&E slides by measuring the greatest thickness, from tumor surface to submucosa, of eight rapamycin-treated tumors and six vehicle-treated tumors from *iLgr5;GLI2A* mice. Average thickness of mucosa in normal antrum (*N* = 7) was also measured, and this value was subtracted from the average thickness of rapamycin-treated or vehicle-treated tumors.

### Tumor allografts

Tumors were incubated 10 minutes in antibiotic solution (1000 units/ml penicillin and 1000μg/ml streptomycin in RPMI medium with 5% FBS), minced into 1mm^2^ pieces, and resuspended in Matrigel at a 1:4 ratio. An 18-gauge needle was used to inject 0.3 ml of suspension subcutaneously into 7 week-old FVB/NJ nude mice (Jackson Laboratory), which were fed doxycycline-containing chow starting 2 days before injection.

### qRT-PCR analysis and statistics

Tissue samples were collected and frozen in RNAlater (Qiagen). Total RNA was extracted using an RNeasy Mini Kit (Qiagen). Two μg of total RNA was transcribed into single-stranded complementary DNA using SuperScript III Reverse Transcriptase (Invitrogen) in a 20 μl reaction. Two μl of a 1:5 dilution of cDNA was used in a 20 μl qRT-PCR reaction mix containing 10 μl of power SYBR Green PCR Master Mix (Applied Biosystems). Primer sequences are listed in Supplementary Table 3. Real-time PCR was performed in duplicate for each sample using the StepOnePlus Real-Time PCR System (Applied Biosystems), data were analyzed with StepOne software, and normalized to the expression of hypoxanthine guanine phosphoribosyltransferase. Results are expressed as relative increase in the level of gene expression, compared to controls, using the threshold cycle (Ct) slope method. Statistical significance was determined using the two-tailed, unpaired t, Mann-Whitney test (nonparametric). Statistical analysis was performed using GraphPad Prism (version 6) software, which calculates exact P values. Data are presented as mean ± SEM, with P values less than 0.05 considered significant.

## SUPPLEMENTARY MATERIAL FIGURES AND TABLES


