# Supplementary material for: Invasive mouse gastric adenocarcinomas arising from Lgr5+ stem cells are dependent on crosstalk between the Hedgehog/GLI2 and mTOR pathways
Source: Oncotarget. 2016 Feb 3;7(9):10255–70. doi: 10.18632/oncotarget.7182 (PMC4891118; doi:10.18632/oncotarget.7182)
Supplement: Supplementary file 1 [file oncotarget-07-10255-s001.pdf]

Invasive mouse gastric adenocarcinomas arising from Lgr5+ stem cells are dependent on crosstalk between the Hedgehog/GLI2 and mTOR pathways

Supplementary Material

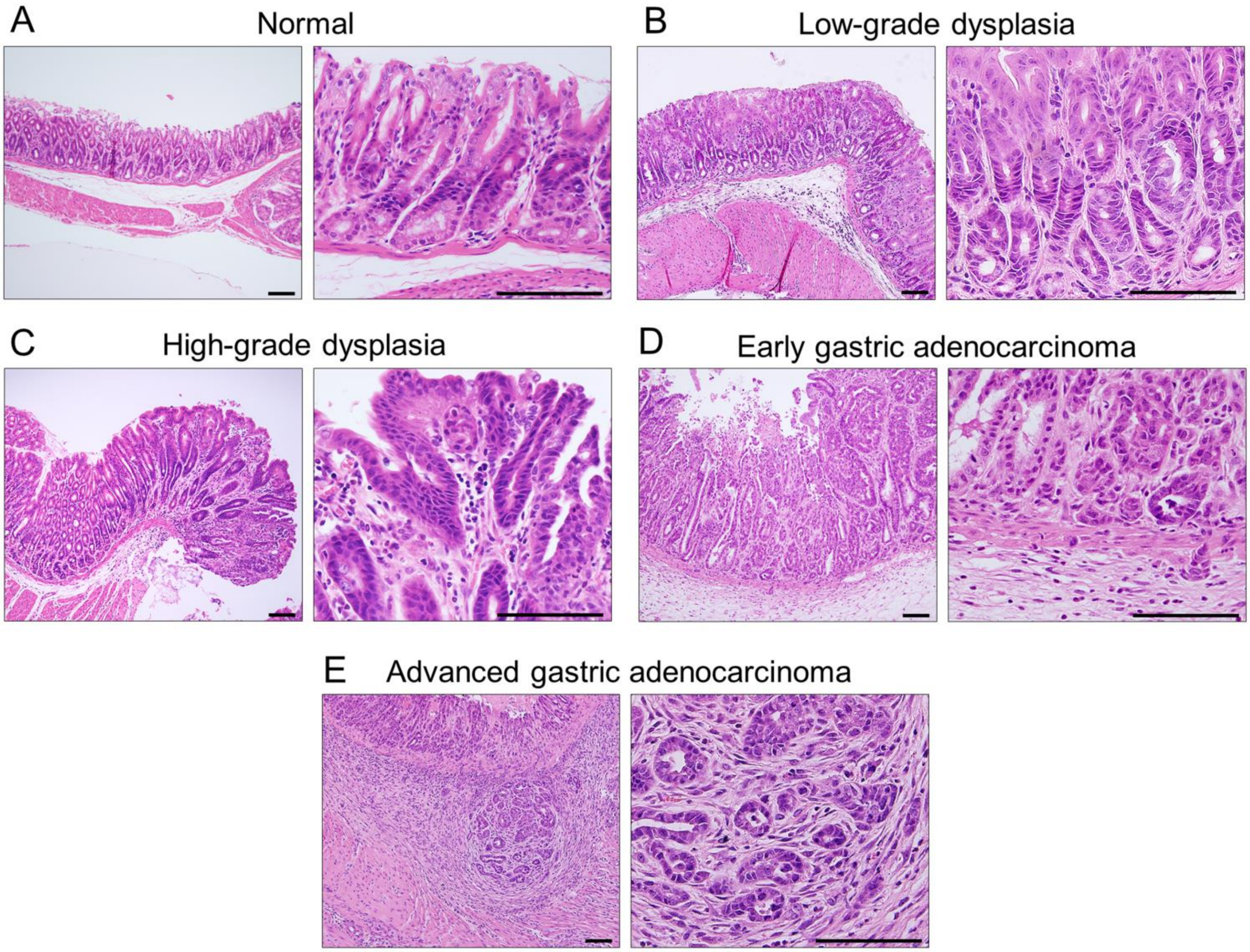

Supplementary Figure 1. Representative histopathology used for scoring of gastric lesions arising in *iLgr5;GLI2A* mice. A) Normal antrum. B) Low-grade dysplasia, showing a slightly modified mucosal architecture, the presence of tubular structures with budding, and glands that are lined by enlarged columnar cells with rounded or ovoid nuclei. C) High-grade dysplasia, showing architectural distortion of the gastric glands with papillary infolding, crypt lengthening, loss of mucin-containing cells, and prominent cellular atypia. D) Early gastric carcinoma, showing a moderately well-differentiated adenocarcinoma with irregular tubules varying in diameter. Tumor cells are columnar or cuboidal with cytological atypia varying from low to high-grade, with invasion into the lamina propria and submucosa. E) Advanced gastric carcinoma, showing moderately-well differentiated adenocarcinomas invading the mucularis propria. Scale bars: 100 μm.

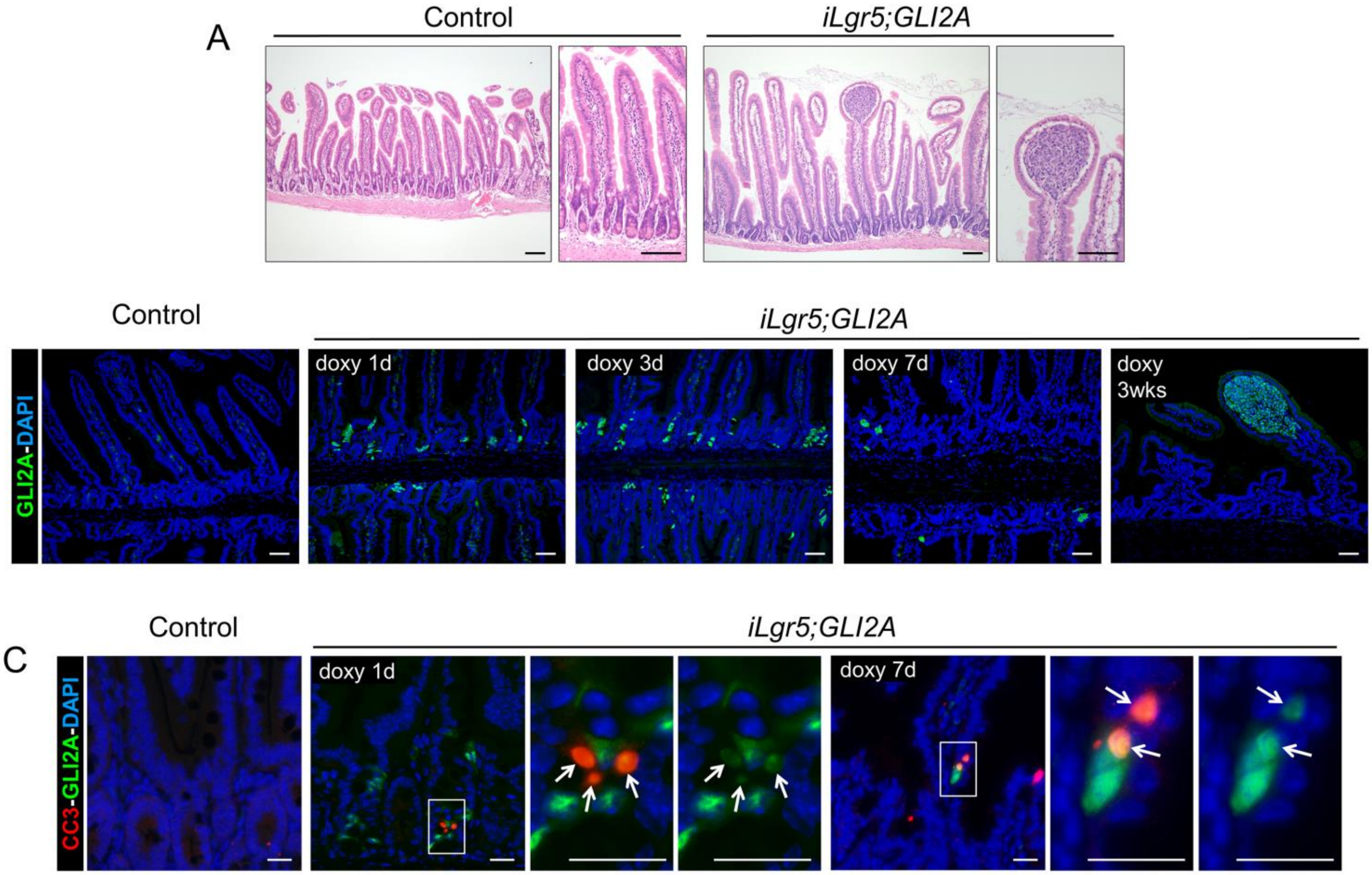

Supplementary Figure 2. Lgr5-expressing stem cells in intestine do not yield epithelial tumors in response to GLI2A. A) Intestine collected after 3 weeks of GLI2A induction in *iLgr5;GLI2A* mice did not contain epithelial tumors, although tumor-like collections of mesenchymal cells were detected in tips of some villi. B) Transient appearance of GLI2A-expressing epithelial cells detected by immunostaining within intestinal crypts. At 3 weeks, GLI2A-expressing cells were limited to the mesenchymal aggregates in villi. C) GLI2A-expressing epithelial cells in intestine undergo apoptosis at early time points, based on co-localization of GLI2A and cleaved caspase 3 (CC3) (arrows). Scale bars: 100 μm.

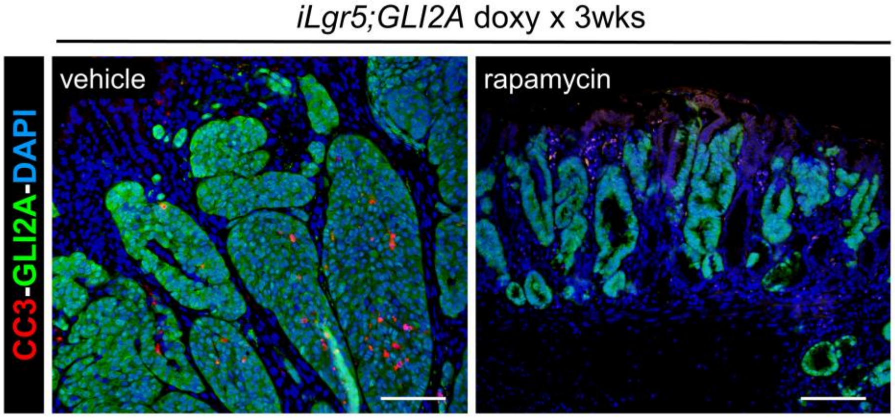

Supplementary Figure 3. Reduced cell death in GLI2A-driven tumors from mice treated with rapamycin for three weeks. Inhibition of mTOR signaling with rapamycin led to reduced expression of the apoptosis marker cleaved caspase 3 (CC3) within tumor nodules. Scale bars: 100 μm.

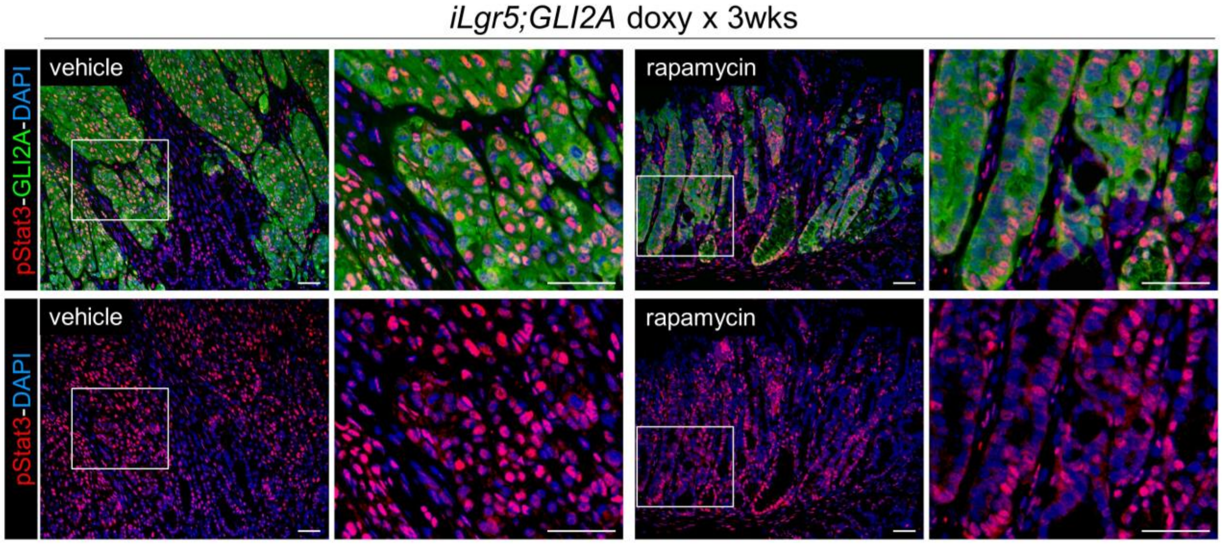

Supplementary Figure 4. Rapamycin treatment does not extinguish Stat3 activation in GLI2A-driven gastric cancers. Double-immunostaining for pStat3 and GLI2A in tumors revealed similarly widespread expression of pStat3 in tumors from vehicle- and rapamycin-treated *iLgr5;GLI2A* mice. Scale bars: 50 µm.

Supplementary Table 1. Primary Antibodies for Immunostaining

Antibody	Catalog#	Source
Myc-Tag (9B11)	2276	Cell Signaling
c-Myc (goat polyclonal)	NB600-335	Novus Biologicals
Ki-67 (clone SP6)	RM-9106	Thermo Scientific
Cleaved caspase 3 (Asp175)	9661	Cell Signaling
phospho-Histone H2A.X (Ser139) (20E3)	9718	Cell Signaling
MUC5AC	MS-145-P0	Thermo Scientific
Spasmolytic polypeptide (GE16C)	ab49536	Abcam
Actin, smooth muscle Ab-1 (Clone 1A4)	MS-113-P0	Thermo Scientific
CD45	14-0451	eBioscience
CD3	A0452	DakoCytomation
F4/80	14-4801	eBioscience
Myeloperoxidase	A0398	DakoCytomation
phospho-Stat3 (Tyr705) (D3A7)	9145	Cell Signaling
phospho-S6 Ribosomal Protein (Ser240/244)	2215	Cell Signaling
phospho-p44/42 MAPK (Erk1/2) (Thr202/Tyr204)	9101	Cell Signaling
Snail (L70G2)	3895	Cell Signaling
Lef1 (C12A5)	2230	Cell Signaling
E-Cadherin (24E10)	3195	Cell Signaling
Vimentin (D21H3)	5741	Cell Signaling
β-catenin	C7207	Sigma
Keratin 17	NA	Pierre A. Coulombe

Supplementary Table 2. Primary Antibodies for Immunoblotting

Antibody	Catalog#	Source
Myc-Tag (9B11)	2276	Cell Signaling
Akt	9272	Cell Signaling
phospho-Akt (Ser473)	9271	Cell Signaling
S6 Ribosomal Protein (5G10)	2217	Cell Signaling
phospho-S6 Ribosomal Protein (Ser240/244)	2215	Cell Signaling
Stat3	9132	Cell Signaling
phospho-Stat3 (Tyr705) (D3A7)	9145	Cell Signaling
p70 S6 Kinase (49D7)	2708	Cell Signaling
phospho-p70 S6 Kinase (Thr389)	9205	Cell Signaling
phospho-p70 S6 Kinase (Thr421/Ser424)	9204	Cell Signaling
PCNA	RB-9055	Thermo Scientific
β-actin	A5316	Sigma

Supplementary Table 3. Real-Time quantitative PCR primers

mRNA	Forward primer	Reverse primer	Source
Snail1	(+) 5' - CTCTGAAGATGCACATCCGAAGC	(-) 5'- GCAGTGGGAGCAGGAGAATGG	this reference
Snail2	(+) 5' - CGTGCTGCTTCAAGGACACATTAG	(-) 5'- GCGACATTCTGGAGAAGGTTTTGG	this reference
Fsp1	(+) 5' - TTGTGTCCACCTTCCACA	(-) 5'- GCTGTCCAAGTTGCTCAT	this reference
Twist1	(+) 5' - GATTCAGACCCTCAAACTGGCG	(-) 5'- AGACGGAGAAGGCGTAGCTGAG	Origene# MP217798
Zeb1	(+) 5' - CCACTGTGGAGGACCAGAAT	(-) 5'- CTCGTGAGGCCTCTTACCTG	this reference
Hprt	(+) 5' - AGGACCTCTCGAAGTGTTGGATAC	(-) 5'- AACTTGCGCTCATCTTAGGCTTTG	this reference
IL-6	(+) 5' - GAGGATACCACTCCCAACAGACC	(-) 5'- AAGTGCATCATCGTTGTTCATACA	this reference
IL-11	(+) 5' - CTGACGGAGATCACAGTCTGGA	(-) 5'- GGACATCAAGTCTACTCGAAGCC	Origene# MP206741
IL-1β	(+) 5' - CAACCAACAAGTGATATTCTCCATG	(-) 5'- GATCCACACTCTCCAGCTGCA	this reference
TNFα	(+) 5' - CATCTTCTCAAAATTCGAGTGACAA	(-) 5'- TGGGAGTAGACAAGGTACAACCC	this reference
Gli1	(+) 5' - TTGGGATGAAGAAGCAGTTG	(-) 5'- GGAGACAGCATGGCTCACTA	this reference
Ptch1	(+) 5' - CTGCTGTGGTGGTGGTATTC	(-) 5'- GGCTTGTGAAACAGCAGAAA	this reference
Egfr	(+) 5' - GGACTGTGTCTCCTGCCAGAAT	(-) 5'- GGCAGACATTCTGGATGGCACT	Origene# MP204284
Her2	(+) 5' - GACCTCAGTGTCTTCCAGAACC	(-) 5'- TGCGGTGAATGAGAGCCAATCC	this reference
Egf	(+) 5' - ACTGGTGTGACACCAAGAGGTC	(-) 5'- CCACAGGTGATCCTCAAACACG	Origene# MP204282
Areg	(+) 5' - GCAGATACATCGAGAACCTGGAG	(-) 5'- CCTTGTCATCCTCGCTGTGAGT	Origene# MP200616
Ereg	(+) 5' - CAGGCAGTTATCAGCACAACCG	(-) 5'- CATGCAAGCAGTAGCCGTCCAT	Origene# MP20429
Hbegf	(+) 5' - CGGGGAGTGCAGATACCTG	(-) 5'- TTCTCCACTGGTAGAGTCAGC	this reference
Tgfα	(+) 5' - CAGGCTCTGGAGAACAGCACAT	(-) 5'- GACACATGCTGGCTTCTCTTCC	Origene# MP217180
